# The Mental Health Impact of Financial mistreatment: The Mediating Role of Perceived Stress

**DOI:** 10.1093/geroni/igaf122.2046

**Published:** 2025-12-31

**Authors:** Juyoung Park, Yuri Jang

**Affiliations:** Emory University, Georgia, United States; University of Southern California, Los Angeles, California, United States

## Abstract

Financial mistreatment is a public health concern with severe consequences for older adults’ mental well-being. While prior research has established a direct link between financial mistreatment and depressive symptoms, the mechanisms underlying this relationship remain underexplored. Guided by the Stress Appraisal Model, this study examined the mediating role of perceived stress in the association between financial mistreatment and depressive symptoms. Using data from the National Social Life, Health, and Aging Project Round 3 (N = 2,409, Mean age = 75.8, SD = 6.94), we conducted multivariate regression and mediation analyses to examine the dynamics among financial mistreatment, perceived stress, and depressive symptoms. Results demonstrated that financial mistreatment (B [SE] = 1.09 [.22], p < .001) and perceived stress (B [SE] = .54 [.04], p < .001) had significant direct effects on depressive symptoms. We also found a significant indirect effect (B [SE] = .20 [.07], bias-corrected 95% bootstrap confidence interval = 0.06, 0.35), where the mental health impact of financial mistreatment was mediated through perceived stress. These findings suggest that financial mistreatment contributes to depressive symptoms both directly and indirectly by intensifying perceived stress levels. As a critical life stressor, the experience of financial mistreatment may elevate perceived stressfulness, which in turn may lead to depressive symptoms. While prevention efforts are essential, our findings highlight the critical need to expand access to stress management resources and services for those financially mistreated.

